# Effects of Rearing Temperatures on Key Biological Parameters of the Egg Parasitoids *Trichogramma cocoeciae* and *Trichogramma bourarachae* (Hymenoptera: Trichogrammatidae): Implications for Biological Control

**DOI:** 10.3390/insects17050456

**Published:** 2026-04-27

**Authors:** Nihel Ben Saad, Mehdia Fraj, Ramzi Mansour, Anis Zouba, Kaouthar Grissa Lebdi, Sahar Zougari, Ahmed Mahmoud Ismail, Hossam S. El-Beltagi, Saleh Mbark Alturki, Saad N. Al-Kahtani, Mohamed J. Hajjar, Tarek A. Shalaby, Husameldin Mahmoud, Sabrine Attia

**Affiliations:** 1 Laboratory of Bioaggressors and Integrated Pest Management in Agriculture (LR14AGR02), National Agronomic Institute of Tunisia (INAT), University of Carthage, 43 Avenue Charles Nicolle, Cité Mahrajène, Tunis 1082, Tunisia; nihelbensaad@gmail.com (N.B.S.); kaouthar.grissa@inat.ucar.tn (K.G.L.); 2 Laboratory of Diversity, Management and Conservation of Biological Systems (LR18ES06), Faculty of Sciences of Tunis, University of Tunis El Manar, Tunis 2092, Tunisia; frajmehdia@gmail.com; 3 Section of Biological Sciences, ISEP—BG Soukra, University of Carthage, 49 Avenue 13 Août, La Soukra 2036, Tunisia; ramzi_mansour82@yahoo.co.uk; 4 Date Palm Technical Center (CTD), Kebili 4280, Tunisia; aniszoubactd@yahoo.com (A.Z.); sahar.zougari@gmail.com (S.Z.); 5 Pests and Plant Diseases Unit, College of Agricultural and Food Sciences, King Faisal University, Al-Ahsa 31982, Saudi Arabia; 6 Agricultural Biotechnology Department, College of Agricultural and Food Sciences, King Faisal University, Al-Ahsa 31982, Saudi Arabia; helbeltagi@kfu.edu.sa; 7 Department of Arid Land Agriculture, College of Agricultural and Food Sciences, King Faisal University, Al-Ahsa 31982, Saudi Arabia; smturki@kfu.edu.sa (S.M.A.); salkahtani@kfu.edu.sa (S.N.A.-K.); mhajjar@kfu.edu.sa (M.J.H.); tshalaby@kfu.edu.sa (T.A.S.); hkhalaf@kfu.edu.sa (H.M.); 8 Department of Entomology, University of Georgia, 1109 Experiment Street, Griffin, GA 30223, USA

**Keywords:** egg parasitoid, thermal tolerance, emergence, parasitism, longevity, sex ratio, biological control

## Abstract

The field effectiveness of *Trichogramma* parasitoids (Hymenoptera: Trichogrammatidae) against lepidopteran pests is strongly influenced by temperature, which affects their development, survival, parasitism and reproductive performance. Understanding thermal requirements is therefore essential for optimizing mass rearing and release strategies. The present study evaluated the effects of five constant temperatures (25, 30, 33, 35, and 40 °C) on biological parameters of *Trichogramma bourarachae* Pintureau & Babault and two strains (S1 and S2) of *T. cacoeciae* Marchal reared on *Ephestia kuehniella* Zeller eggs. Emergence rates were higher between 25 °C and 33 °C for all tested parasitoids, decreased markedly at 35 °C for *T. cacoeciae*, whereas *T. bourarachae* emergence showed higher tolerance at 35 °C, and no emergence was recorded for all parasitoids at 40 °C. Parasitism capacity was strongly influenced by both temperature and parental thermal history. *Trichogramma bourarachae* exhibited its highest parasitism rate at 25 °C; however, females originating from the parental generation that developed at 30 °C maintained relatively high parasitism rates at elevated temperatures (30 to 35 °C). For *T. cacoeciae* S1, parental development at 30 °C enhanced offspring parasitism over a broader temperature range. Conversely, *T. cacoeciae* S2 achieved maximum parasitism when the parental generation developed at 25 °C, with high parasitism maintained at 25 °C, 30 °C, and 33 °C. At the species level, parasitism was highest between 25 °C and 33 °C, declined at 35 °C, and no parasitism was recorded at 40 °C due to the absence of survival. Within each species, however, strain-specific differences were observed, particularly at 35 °C, indicating variability in thermal tolerance and reproductive performance. Female longevity decreased with increasing temperature in all species and strains. However, individuals originating from parental generation that developed at 30 °C exhibited improved survival when exposed to elevated oviposition temperatures, indicating thermal acclimation. Increasing temperature induced a male-biased sex ratio in *T. bourarachae*, whereas *T. cacoeciae* maintained stable thelytokous reproduction across all treatments. These results emphasize the importance of thermal tolerance and parental thermal history for selecting suitable *Trichogramma* species and strains for mass rearing and field application for biological control under warming climatic conditions.

## Simple Summary

We assessed the effects of five constant rearing temperatures on key biological parameters of *Trichogramma bourarachae* Pintureau & Babault and two strains (S1 and S2) of *T. cacoeciae* Marchal reared on *Ephestia kuehniella* Zeller eggs. Higher emergence rates were observed between 25 °C and 33 °C for all parasitoids. However, at 35 °C, emergence declined in *T. cacoeciae*, whereas *T. bourarachae* showed greater tolerance to elevated temperature. Parasitism rates were significantly influenced by temperature and parental thermal history. *Trichogramma bourarachae* showed maximum parasitism at 25 °C, while parental development at 30 °C improved performance at higher temperatures. In *T. cacoeciae*, optimal parasitism varied between strains depending on parental rearing temperature. Female longevity decreased with increasing temperature, but parasitoids originating from parental generation that developed at 30 °C showed improved survival at higher temperatures. Temperature increase induced a male-biased sex ratio in *T. bourarachae*, while *T. cacoeciae* maintained stable thelytokous reproduction. These results highlight the importance of considering thermal tolerance and parental thermal history for selecting *Trichogramma* strains for mass rearing and biological control purposes.

## 1. Introduction

*Trichogramma* spp. (Hymenoptera: Trichogrammatidae) are among the most widely used egg parasitoids in the biological control of lepidopteran pests worldwide; their success is mainly due to their broad host range and high efficacy against numerous target pests. In addition, they are easily mass-reared under laboratory conditions [1,2,3,4]. Currently, more than ten *Trichogramma* species are commercially or experimentally used in biological control programs against key crop pests, such as *Ectomyelois ceratoniae* (Zeller) (Lepidoptera: Pyralidae) [5,6,7,8], *Tuta absoluta* (Meyrick) (Lepidoptera: Gelechiidae) [9,10,11,12], *Spodoptera frugiperda* Smith (Lepidoptera: Noctuidae) [13,14,15,16], *Helicoverpa armigera* Hübner (Lepidoptera: Noctuidae) [4,17,18,19], and *Ostrinia nubilalis* Hübner (Lepidoptera: Crambidae) [2,20,21,22]. These egg parasitoids have been released in various agroecosystems, including cotton, maize, vegetables, and tree fruits, representing one of the most successful examples of augmentative biological control [23,24,25,26]. In Tunisia, the use of *Trichogramma* wasps is becoming increasingly prominent, as they are now officially integrated into national integrated pest management programs targeting major economic pests, such as the carob moth, *E. ceratoniae* [7,8], and the invasive South American tomato pinworm, *T. absoluta* [27,28,29]. However, despite their recognized biocontrol potential, the field performance of *Trichogramma* spp. often remains inconsistent due to local environmental constraints, especially temperature extremes and pronounced thermal fluctuations characterizing arid and semi-arid regions. A crucial step in developing an effective biological control program using *Trichogramma* involves selecting the most potent species or strain for managing the target pest and maintaining damage below an economically acceptable threshold [30,31]. Nevertheless, the efficacy of a parasitoid depends not only on its ability to parasitize hosts but also on its tolerance and adaptation to the thermal conditions of the release area [32,33,34]. A comprehensive understanding of the environmental factors, especially temperature, influencing reproductive performance of *Trichogramma* spp. under field conditions is essential for successful inundative releases. The ability to sustain high reproductive potential and survival under suboptimal temperatures is therefore a key factor for successful establishment and control efficiency [35,36,37,38]. Climate change has caused significant increases in global temperatures, disrupting ecosystem dynamics [39,40]. These effects are particularly severe for ectothermic organisms, as temperature governs nearly all aspects of insect biology, including development, survival, fecundity, and longevity [3,39,41,42,43,44].

Temperature affects development through two principal pathways: (i) directly modulating metabolic activity and growth rate, which generally increase within an optimal thermal range [45,46], and (ii) acting alone or in interaction with photoperiod as a key environmental cue regulating diapause induction [47]. Parasitoids, including *Trichogramma*, are highly sensitive to temperature throughout all life stages. High temperatures can accelerate development but often reduce longevity [37,48,49], whereas low temperatures tend to prolong development and increase mortality [32,37,38,50]. In addition to mean temperature, short-term thermal extremes and fluctuating regimes can strongly affect parasitoid performance. For example, heat or cold shocks can alter diapause incidence in *Trichogramma telengai* Sorokina [51], while fluctuating temperatures influence both diapause induction and developmental rates [52]. Similarly, heat stress during development may reduce emergence, longevity, and fecundity in related species such as *Trichogramma bactrae* Nagaraja [36,53,54].

Temperature also influences size and morphology, which may affect flight ability, host-searching behavior, and parasitism efficiency in the field [55,56]. Moreover, thermal conditions experienced during development can induce phenotypic plasticity in later generations. This transgenerational thermal effect, documented in parasitoids such as *Aphidius colemani* Viereck [57], shows that parental rearing temperature can influence offspring fecundity, sex ratio, and heat tolerance, which are critical for mass-rearing and field establishment of *Trichogramma* populations [58,59]. Such carry-over effects highlight the importance of strain selection to ensure stable performance under fluctuating field temperatures. For example, difference in thermal tolerance and parasitism capacity have been reported between strains of *T. cacoeciae*, with the Tunisian strain showing better adaptation and higher parasitism rates under high temperature conditions compared to the Italian strain [60]. Therefore, parental rearing temperature was explicitly incorporated into the experimental framework of the present study to evaluate its influence on key biological parameters. Understanding how temperature changes influence life-history traits of parasitoids is essential for predicting population dynamics and optimizing biological control applications [61]. Moreover, the development of efficient storage techniques is crucial for ensuring a continuous supply of parasitoids for mass production and field release. Indeed, pupal stages of different strains of *Trichogramma chilonis* Ishii stored at low temperatures (5 and 10 °C) exhibited marked strain-specific differences in their ability to maintain developmental arrest, emergence rate, and post-emergence survival, with some strains remaining viable for up to 50–60 days under optimal conditions [62].

The egg parasitoids *Trichogramma bourarachae* Pintureau & Babault and *T. cacoeciae* Marchal, two commercially important biocontrol candidates, have been successfully used against key lepidopteran pests across various agricultural systems worldwide. Although aspects of the life cycle of either *T. bourarachae* or *T. cacoeciae* have already been studied [33,63,64,65], different strains/ecotypes of these parasitoids from diverse geographic regions exhibit variable biological traits, which could affect their success in biological control programs [2,33,66]. Similarly, species-and strain-specific differences in emergence rate and longevity under cold storage conditions have already been reported, highlighting variability in physiological tolerance and performance among *Trichogramma* spp. [67].

In this context, comparative studies on thermal sensitivity among different *Trichogramma* species and strains are crucial for identifying candidates best suited to Mediterranean climatic conditions, where summer temperatures often exceed 40 °C.

Despite extensive research on temperature-dependent performance in *Trichogramma* spp., the interactive effects of parental thermal history and interspecific/strain variability under high-temperature conditions remain poorly understood. This study addresses this gap by providing a comparative analysis of two species and two strains across a wide thermal range, with a particular focus on transgenerational thermal effects relevant to biological control under climate warming.

The aim of the present study was to assess the effects of five rearing temperatures (25, 30, 33, 35, and 40 °C) on key biological parameters including pre-adult development, emergence rate, parasitism rate, survival (longevity), and sex ratio of *T. bourarachae* and two strains of *T. cacoeciae*. The present research would provide insights into how these parasitoids respond to climate change, which would optimize mass-rearing strategies and the timing of augmentative releases against target lepidopteran pests throughout Mediterranean agro-ecosystems.

## 2. Materials and Methods

### 2.1. Egg Parasitoid Rearing

*Trichogramma cacoeciae* is a thelytokous parasitoid that reproduces independently of *Wolbachia* symbiotic bacteria [68]. *Trichogramma cacoeciae* strain 1 (S1) was introduced as a local population in 2020 by collecting parasitoid wasps from host baits (sentinel eggs of *Ephestia kuehniella* Zeller (Lepidoptera: Pyralidae) in date palm orchards located in the southwest of Tunisia. However, strain 2 (S2) of *T. cacoeciae* was established in 2021 by collecting wasps from parasitized eggs of the carob moth *E. ceratoniae* in pomegranate orchards in southeastern Tunisia. The two strains of *Trichogramma cacoeciae* differ in their geographic origin, host species of origin, agroecosystem, and year of collection. Additionally, one arrhenotokous species, *T. bourarachae*, was initially collected from a date palm orchard using host baits (sentinel eggs of *E. kuehniella*) in the Tozeur oases, located in the southwest of Tunisia. In 2022, these two strains of *T. cacoeciae* and *T. bourarachae* were introduced in the laboratory of Date Palm Technical Center (CTD) in Kebili, Tunisia, for research and conservation purposes. The *Trichogramma* species studied were kept on ultraviolet-sterilized eggs of *E. kuehniella* in a climate-controlled chamber (25 ± 2 °C temperature, 70 ± 5% relative humidity (RH), and 16 h:8 h (L:D) photoperiod). Factitious host eggs were obtained from a culture of *E. kuehniella* reared on a standard diet composed of wheat flour, prepared according to the methodology described by Cerutti et al. [69].

### 2.2. Effect of Rearing Temperatures on Trichogramma Parasitoids

The experimental protocol design used in this study was similar to that described by Pizzol et al. [33] with slight modifications. In our study, five temperature treatments were evaluated (25, 30, 33, 35, and 40 ± 2 °C) instead of the four temperatures (15–30 °C) used in the reference protocol. In addition, multiple *Trichogramma* species and strains were tested rather than a single species. The oviposition period was extended to 24 h instead of 2 h to allow females to fully express their reproductive potential and to better reflect mass-rearing and biological control conditions. Furthermore, additional biological parameters, including daily mortality and sex ratio, were assessed.

### 2.3. Effect of Temperature on the First Generation (G1)

Females of each *Trichogramma* species and strains were allowed to oviposit at 25, 30, 33, 35, and 40 °C (±2 °C) in climatic chambers. For each treatment, 30 newly emerged females (<24-h-old) of each species and strain were individually isolated in glass vials (7.5 cm long × 1 cm diameter) to provide sufficient replication for statistical analysis and to account for individual variability in key biological traits. Each female parasitoid was provided with a drop of honey as food and supplied with fresh eggs of *E. kuehniella* as host (800 ± 50 one-day-old eggs sterilized with UV light) glued on a cardboard card (1 cm × 5 cm). This number of host eggs was provided in excess to prevent host limitation and to ensure accurate and comparable estimates of parasitism across treatments. Each isolated female represented a replicate. After 24 h, all females were removed and discarded from their vials using a thin brush, and the parasitized eggs were incubated at the respective pre-adult temperatures (25, 30, 33, 35, and 40 ± 2 °C). For each *Trichogramma* species and strain, this procedure provided five first-generation cohorts, hereafter referred to as G1–25, G1–30, G1–33, G1–35, and G1–40. The effect of temperature on parasitoids was assessed by recording the emergence rate, calculated as recommended by Van Driesche [70], using the following equation:

Emergence percentage = Number of eggs with emergence holes/Total number of eggs × 100

### 2.4. Effect of Temperature on the Second Generation (G2)

Females obtained from the above experiment were randomly selected at each of the tested pre-adult temperatures; only for the batch at 40 °C were no females selected, as no adult parasitoids emerged at this temperature in the G1 generation. Then 150 newly emerged females (<24-h-old) of each species and strain were collected from each of the four first-generation batches (G1-25, G1-30, G1-33 and G1-35); groups of 30 females were exposed during the oviposition period to each of the tested temperatures (25, 30, 33, 35, and 40 °C). Each treatment combination consisted of 30 individual females, each representing one replicate. Each female was individually isolated in glass vials (7.5 cm long × 1 cm diameter), fed with a drop of honey, and offered an egg card with 500 ± 50 one-day-old sterilized *E. kuehniella* eggs (6 cm × 0.9 cm). Each isolated female represented a replicate. In this way, females originating from 25 °C during G1 were tested at all five temperatures during G2, and the same procedure was followed for females that had developed at 30, 33, and 35 °C in G1. This gives 20 combinations for the second generation (G2). After exposing females to the host eggs for 7 days, they were removed. Three biological parameters were calculated: (i) parasitism rate was defined as the total number of successfully parasitized eggs by females over the 7 days; (ii) female mortality was monitored daily during the 7 days and the mortality was expressed as the cumulative number of dead females per day, and (iii) sex ratio was estimated using the following equation:

Female percentage = Number of females/Total number × 100

### 2.5. Data Analysis

All statistical analyses were performed using R version 4.5.0 [71]. Data were transformed before analysis to stabilize the variance [72]: emergence rate, sex ratio, and mortality by arcsin square root (arcsin √x) and fecundity by inverse (1/x). A generalized linear model (GLM binomial or Poisson) was then used to assess the effects of strain, temperature (G1), temperature (G2), and their interactions on the measured biological traits of the three studied strains. Overdispersion was assessed by comparing residual deviance to degrees of freedom to confirm the suitability of binomial and Poisson error structures. Binomial error distributions were used for proportional response variables (emergence rate, sex ratio, and mortality), whereas a Poisson error distribution was applied for count data (parasitism rate). ANOVA (Type III) on the GLM was used to evaluate the significance of each factor and interaction, based on the comparison of model deviance (likelihood ratio test).

## 3. Results

### 3.1. Emergence Rate in the First Generation (G1)

**Figure 1:**
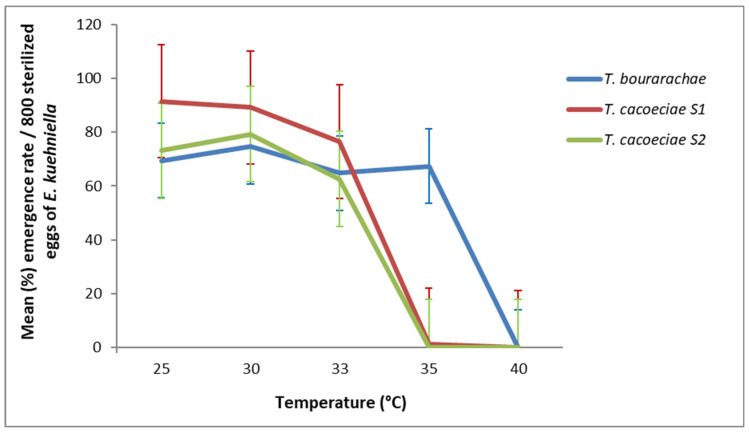
Mean emergence rate (% ±SD) of *T. bourarachae* and *T. cacoeciae* strains S1 and S2 from sterilized *E. kuehniella* eggs at different temperatures applied during the first generation (G1). Error bars represent standard deviation. Each treatment was replicated 30 times (*n* = 30 females per strain per temperature).

The generalized linear model (GLM) showed that both species and strains, as well as temperature, significantly affected the emergence rate of first-generation *Trichogramma* (strain: χ^2^ = 226.1, df = 2, *p* < 0.001; temperature: χ^2^ = 727.9, df = 1, *p* < 0.001). The interaction between strain and temperature was also significant (χ^2^ = 230.4, df = 2, *p* < 0.001). This interaction suggests that strain performance is temperature-dependent, emphasizing the need to select strains with greater tolerance to elevated temperatures for effective biological control in arid and semi-arid regions. All tested species successfully completed development and emerged as adults at 25 °C to 33 °C (Figure 1), whereas no adults emerged at 40 °C. At 25 °C, the two strains of *T. cacoeciae* (S1: 91.5%; S2: 73.2%) showed higher emergence rates than *T. bourarachae* (69.4%). At 35 °C, this trend reversed: *T. bourarachae* achieved the highest emergence (67.4%), while the emergence of *T. cacoeciae* declined sharply (S1: 1.1%; S2: 0%). This pattern clearly indicates a greater thermal tolerance of *T. bourarachae*, suggesting its potential suitability for biological control programs under high-temperature environments. These results indicate marked thermal sensitivity differences among strains and species, which are critical for optimizing biological control applications.

### 3.2. Parasitism Rate in the Second Generation (G2)

The number of *E. kuehniella* eggs parasitized by the two *Trichogramma* species was significantly influenced by strain factor (χ^2^ = 0.002, df = 2, *p* = 0.002). Moreover, fecundity varied significantly depending on the developmental temperature of the females (G1), the temperature during oviposition and later development of their offspring (G2) (χ^2^ = 0.009, df = 3, *p* < 0.001; and χ^2^ = 0.015, df = 4, *p* < 0.001, respectively). No significant interaction was observed between strain and temperature at either generation (G1: *p* = 0.903; G2: *p* = 0.872), indicating that the effects of temperature on parasitism were consistent across strains. In other words, temperature and strain influenced parasitism rates independently, with no evidence that temperature effects differed among strains. Overall, these results indicate that the strain factor and both parental and offspring developmental temperatures are the dominant drivers of parasitism rate variation, while interactions between strain and temperature had little effect.

**Figure 2:**
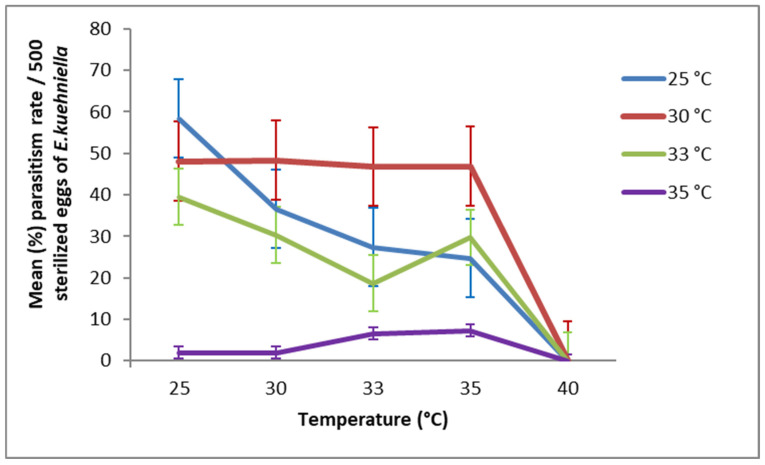
Effect of temperature during the second generation (G2) on the parasitism rate (mean ± SD) of *T. bourarachae* originating from the first generation (G1). Values represent total parasitism over 7 days.

*Trichogramma bourarachae* exhibited optimal parasitism at 25 °C (58.3%) (Figure 2). Females that developed at 30 °C showed relatively high parasitism rates across tested temperatures, reaching 48.35%, 46.79%, and 46.87% at 30 °C, 33 °C, and 35 °C, respectively. In contrast, those developed at 35 °C during G1 showed very low parasitism rates, ranging from 1.9% to 7.3%.

**Figure 3:**
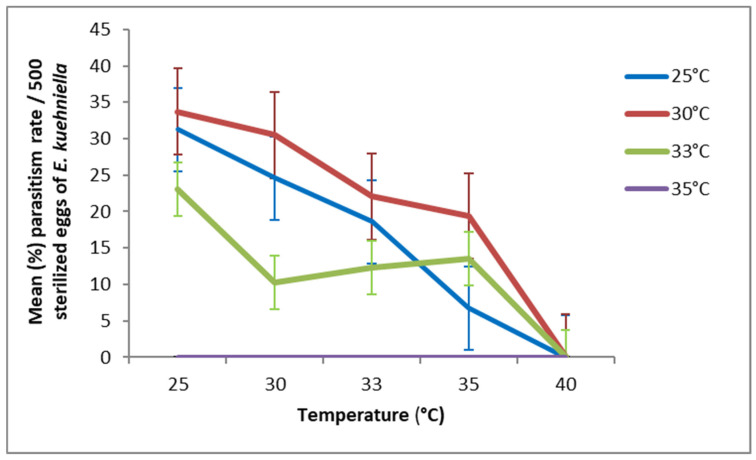
Effect of temperature during the second generation (G2) on the parasitism rate (mean ± SD) of *T. cacoeciae* strain S1 originating from the first generation (G1).

**Figure 4:**
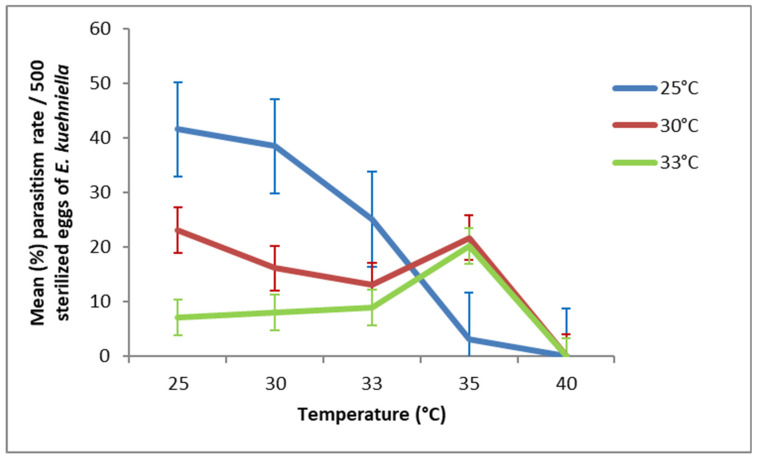
Effect of temperature during the second generation (G2) on the parasitism rate (mean ± SD) of *T. cacoeciae* strain S2 originating from the first generation (G1).

Regarding *T. cacoeciae* S1, females that developed at 25 °C or 30 °C in G1 exhibited a high parasitism rate when ovipositing at 25, 30, or 33 °C (Figure 3). Concerning *T. cacoeciae* S2, the maximum parasitism rate was observed when G1 developed at 25 °C and oviposition occurred at 25, 30, or 33 °C (Figure 4). At 35 °C, parasitism increased only when G1 development occurred at 30 °C or 33 °C, reaching 19.38% and 13.54% in S1, and 21.14% and 20.15% in S2, respectively.

### 3.3. Sex Ratio

**Figure 5:**
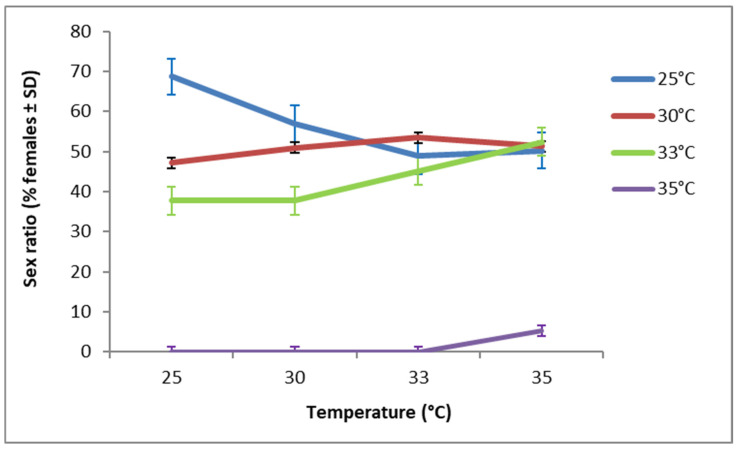
Sex ratio of *T. bourarachae* originating from the first generation (G1) and allowed to oviposit under different temperature regimes.

*Trichogramma cacoeciae* individuals (S1 and S2) did not produce any males under the tested temperature regimes, confirming that reproduction is consistently thelytokous and stable across the entire thermal range tested. However, in *T. bourarachae*, both the developmental temperature of females (G1) and the temperature during oviposition and offspring development (G2) had a highly significant effect on the sex ratio (χ^2^ = 4.104, df = 3, *p* < 0.001; χ^2^ = 4.950, df = 4, *p* < 0.001, respectively). When pre-adult development occurred at 33 °C, the proportion of *T. bourarachae* females increased from 37.74% to 52.37% as the temperature during the egg-laying period (G2) increased from 25 °C to 35 °C (Figure 5). In contrast, when G1 development occurred at 30 °C, the *T. bourarachae* female proportion remained relatively stable at approximately 50% across all temperatures (25, 30, 33, and 35 °C) applied during G2. No *T. bourarachae* females emerged when the first generation developed at 35 °C and eggs were laid at 25 °C, 30 °C, or 33 °C, resulting exclusively in males. However, when the G2 developed at 35 °C, the proportion of *T. bourarachae* females showed a slight increase (5.36%).

### 3.4. Mortality

**Figure 6:**
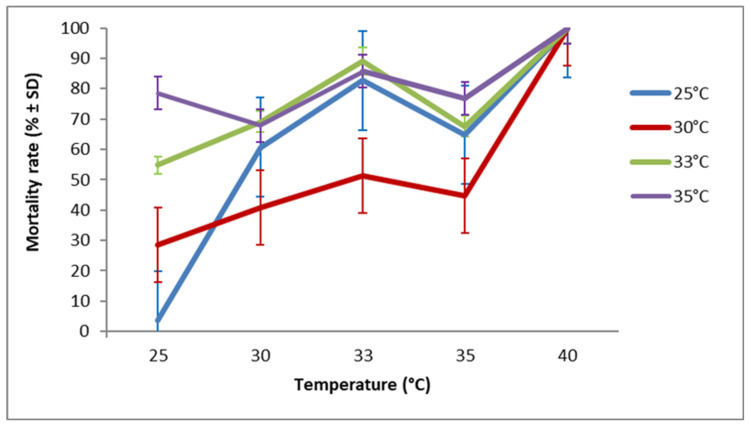
Mortality rate (after 7 days of exposure to host eggs) of *T. bourarachae* originating from the G1 generation and exposed to different temperature regimes.

**Figure 7:**
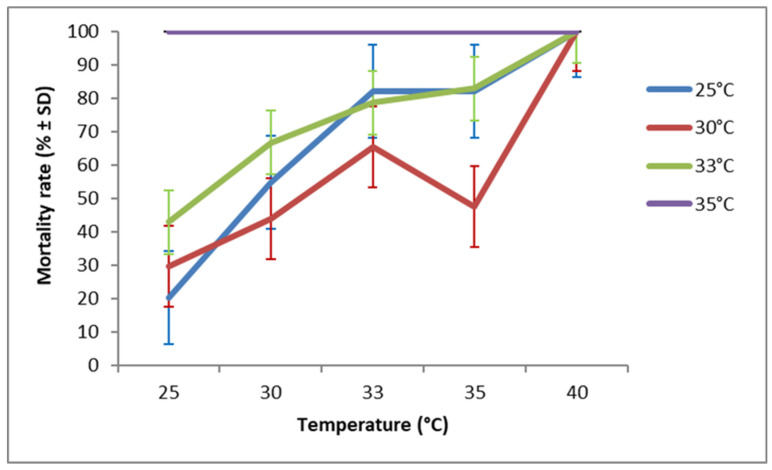
Mortality rate (after 7 days of exposure to host eggs) of *T. cacoeciae* S1 originating from the G1 generation and exposed to different temperature regimes.

**Figure 8:**
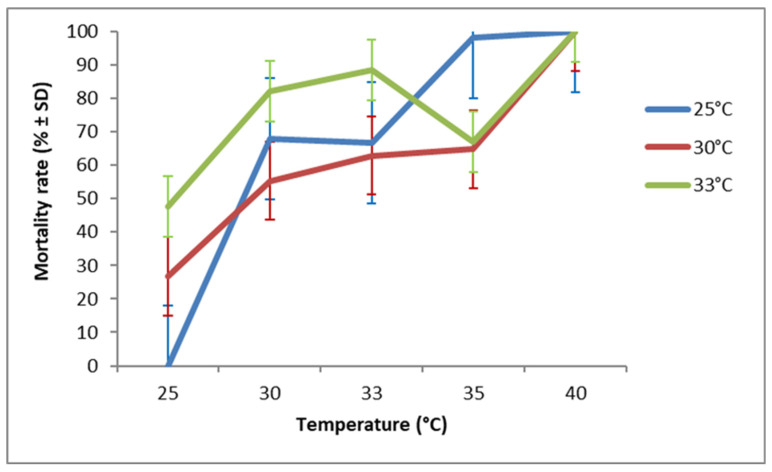
Mortality rate (after 7 days of exposure to host eggs) of *T. cacoeciae* S2 originating from the G1 generation and exposed to different temperature regimes.

Females from different tested strains died at different rates according to their temperature of development during G1 (χ^2^ = 0.713, df = 1, *p* < 0.001), and an even stronger effect was observed for the temperature of the egg-laying period (G2) (χ^2^ = 4.372, df = 1, *p* < 0.001). However, female mortality rate during the 7 days of the egg-laying period did not differ significantly between all tested *Trichogramma* species (χ^2^ = 0.195, df = 2, *p* = 0.185). Furthermore, there was no significant interaction revealed between strain and either temperature factors (*p* > 0.3). The highest mortality was observed at 40 °C for all tested *Trichogramma* species. Overall, *T. bourarachae* (Figure 6) and *T. cacoeciae* S1 (Figure 7) exhibited higher mortality rates when females developed during G1 at 33 °C and 35 °C, whereas *T. cacoeciae* S2 (Figure 8) showed the lowest mortality rates when females developed at 30 °C.

## 4. Discussion

It has always been proven that field performance in *Trichogramma* spp. parasitoids against lepidopteran pests mainly depends on successful laboratory mass rearing, which could be influenced by factors such as artificial diet and host egg quality, storage methods, temperature, relative humidity and photoperiod [3,73]. Our results indicate that *T. cacoeciae* is highly sensitive to high temperatures, with emergence sharply declining above 35 °C, whereas *T. bourarachae* demonstrates greater thermal tolerance. These differences suggest species-specific thermal thresholds that may shape their seasonal activity and suitability for biological control under varying climatic conditions. Exposure to 40 °C resulted in complete failure of adult emergence for both species, highlighting the upper thermal limits for successful parasitism and reproduction. Such findings provide insights into the resilience of these parasitoids under extreme temperature events and can inform predictions of their performance in changing environments. Based on these findings, the upper temperature limit for adult emergence was 35 °C for *T. cacoeciae* (S2) and 40 °C for *T. bourarachae* and *T. cacoeciae* S1, as there was no emergence above these temperatures. These results are consistent with previous studies where the development rate of *Trichogramma brassicae* Bezdenko (Hymenoptera: Trichogrammatidae) declined at 32 °C and 35 °C with an upper threshold between 36 °C and 40 °C [42,61,74,75,76]. Similarly, Schöller and Hassan [77] reported that eggs parasitized by *Trichogramma evanescens* Westwood (Hymenoptera: Trichogrammatidae) became black, but no progeny emerged. The results from the current and previous studies indicate that upper temperature thresholds should be studied carefully before field release for effective pest management.

Females of *T. bourarachae* and *T. cacoeciae* S1 that developed during the first generation at 30 °C exhibited the highest parasitism rates across all tested temperature regimes, whereas *T. cacoeciae* S2 exhibited optimal parasitism when the G1 wasps developed at 25 °C and their offspring (G2) developed following oviposition at 25, 30, or 33 °C. In contrast, when the egg-laying occurred at 35 °C, the wasps that developed during G1 at 30 or 33 °C performed more efficiently than those reared at the other temperature. Across all tested *Trichogramma* species and strains, our experiments revealed that parasitism rate reached its peak at 25 °C, regardless of the temperature at which the first generation was reared. These results are consistent with the majority of previous studies, which reported that 25 °C represents the optimal thermal regime for achieving the highest parasitism performance [33,35,42,77,78,79]. In summary, although 25 °C was the optimal temperature for parasitism across all rearing regimes, developmental exposure to temperature between 30 and 33 °C enhanced the parasitoids’ thermal tolerance and contributed to improved performance under heat stress conditions. This suggests that temperatures experienced by the initial generations of wasps may influence the parasitism efficiency of the next generation. The relatively higher parasitism performance observed for *T. bourarachae* and *T. cacoeciae* S1 at elevated temperatures may reflect prior acclimation or adaptive responses to the challenging environmental conditions characterizing southern Tunisia. However, this interpretation should be considered as a working hypothesis that would require further investigation to confirm local adaptation. Our findings are consistent with previous studies reporting that the behavior of *Trichogramma* species and strains is closely linked to the ecological conditions of their native habitats [33,38,77]. However, at 40 °C, parasitization activity ceased entirely for all *Trichogramma* individuals, as no eggs were parasitized. This suggests that 40 °C could be the upper thermal limit for parasitism, beyond which the parasitoids are unable to maintain their reproductive activity. Similar findings have been found in other *Trichogramma* species, for which upper developmental thresholds ranged from 35 to 46 °C, depending on the species or strain, confirming interspecific variability in thermal tolerance [67]. These findings reinforce the idea that exposure to extreme temperatures can critically constrain the effectiveness of *Trichogramma* parasitoids in biological control programs, particularly under field conditions characterized by heatwaves or prolonged high-temperature periods.

Such thermal inhibition is consistent with the view that extreme heat stress compromises host seeking behavior and parasitism capacity, thereby reducing the biological control potential of *Trichogramma* parasitoids under excessively high temperature [49,80].

Based on our findings, rearing *Trichogramma* spp. at 30–33 °C can be considered optimal when releases are expected at 35 °C or above, as parasitism rates were significantly higher than at 25 °C or 35 °C during the first generation. However, potential trade-offs, such as effects on adult longevity or other fitness traits, should be considered when applying this recommendation. These results are consistent with those reported by Negahban et al. [76] and provide a basis for further field validation where the optimal temperature range for *T. brassicae* was between 30 °C and 33 °C. Similarly, Pizzol et al. [33] showed that a Tunisian strain of *T. cacoeciae* females that developed at 30 °C during the first generation (G1) exhibited high fecundity during oviposition, with their offspring (G2) completing development at 30 °C. Comparable findings were reported by Kalyebi et al. [81] who observed that parasitism rates were highest at 25 °C and 30 °C for six indigenous Kenyan *Trichogramma* egg parasitoid species.

Emergence and parasitism rates were found to be inversely related to temperature, a pattern also reported for *T. chilonis* and *T. poliae* Nagaraja (Hymenoptera: Trichogrammatidae) when exposed to variable temperature ranging from 20 °C to 40 °C through daily short-term heat shocks applied to both immature progeny and egg-laying females [80]. As reported by Alloui-Griza et al. [49], no progeny of *T. cacoeciae* emerged when developed at 40 °C due to the mortality of parasitoids. Several studies demonstrated that, within the thermal tolerance range, emergence and parasitism rates of *Trichogramma* spp. declined with increasing temperature [3,82,83]; consistent with these findings, our results showed that *T. cacoeciae* and *T. bourarachae* followed the same trend.

Our findings revealed an inverse relationship between temperature and adult longevity, where adult longevity decreased as the temperature increased from 25 °C to 40 °C. This result is consistent with a previous study on other *Trichogramma* species, *T. evanescens* and *T. cacoeciae* [77]. In the present study, female mortality after seven days of oviposition was lowest at 25 °C, intermediate at 30–35 °C, and highest at 40 °C. Overall, females from all tested *Trichogramma* species that developed at 30 °C (during G1) showed a relatively low mortality rate when allowed to oviposit at higher temperatures ranging from 30 °C to 35 °C. Pizzol et al. [33], working on two strains (Tunisian and French) of *T. cacoeciae*, reported that female mortality varied depending on the developmental temperature during G1. According to the same authors, the French strain exhibited higher mortality when females had developed at 15 or 30 °C, whereas the Tunisian strain suffered higher mortality when development occurred at 15 °C or 20 °C.

Temperature is a key factor that strongly affects the sex ratio in *Trichogramma* spp. [76,80]. Our experiments revealed that the sex ratio of *T. cacoeciae* (S1 and S2) remained unchanged across all tested temperatures as 100% females were generated. This finding aligns with earlier studies [33,84] that documented the stability of the sex ratio of *T. cacoeciae* because thelytoky was not induced by the endosymbiotic bacteria (*Wolbachia* infection). It appeared that *T. cacoeciae* wasps genetically determined by themselves. However, a female-biased sex ratio was observed in *T. bourarachae* at 25 °C, 30 °C and 33 °C, indicating that moderate temperatures favor female production. In contrast, when preadult development occurred at 35 °C, the proportion of male offspring exceeded that of females. This finding is consistent with Harrison et al. [48], who reported a male-biased sex ratio in *T. pretiosum* Riley (Hymenoptera: Trichogrammatidae) at 35 °C. It is crucial to understand how temperature affects the developmental biology of certain *Trichogramma* species or strains to establish successful mass rearing protocols for biological control programs [85]. In the Mediterranean region, such as Tunisia, the growing season (from May to late September) is characterized by extremely hot and dry conditions, with temperatures frequently exceeding 40 °C in summer. Temperature is a key limiting factor in this region, influencing the activity of *Trichogramma* parasitoids and, consequently, the overall effectiveness of mass releases [8]. In this context, Cherif et al. [60] demonstrated that temperature, often in interaction with relative humidity, significantly affected the life- history traits of *T. cacoeciae*, including parasitism and emergence rate, with elevated temperatures generally leading to a decline in overall performance. Notably, some strains of *T. cacoeciae* may exhibit local adaptation, as evidenced by the Tunisian strain, which has been shown to be better adapted to higher temperatures compared with the Italian strain.

The current study provides essential information on how rearing and oviposition temperatures affect the biological performance of two *Trichogramma* species, highlighting the importance of carefully selecting thermotolerant strains suited to local climate conditions. These findings will support the further development of optimized mass rearing strategies for ensuring more effective, climate-resilient and sustainable biological control programs throughout Mediterranean agro-ecosystems.

## 5. Conclusions

This study highlights the critical influence of temperature on the performance of key *Trichogramma* species and strains. Moderate temperatures (30 to 33 °C) were optimal for emergence and parasitism, whereas exposure to extreme heat (≥40 °C) significantly reduced survival and parasitic activity. These results provide practical guidance for the mass-rearing and field deployment of *Trichogramma parasitoids*, suggesting that maintaining moderate rearing conditions can enhance biological control efficacy under high-temperature environments. The differential responses among species and strains suggest that their ecological origin has shaped strong thermal specialization. The enhanced thermotolerance of *T. bourarachae* and *T. cacoeciae* (S1) reflects adaptive evolution to arid and semi-arid habitats typical of southern Tunisia.

Temperature also influenced adult longevity and sex ratio. Increasing temperature reduced female lifespan and shifted the sex ratio toward males in *T. bourarachae*, whereas *T. cacoeciae* maintained stable thelytokous reproduction (100% females), confirming its genetically determined parthenogenesis rather than *Wolbachia*-induced thelytoky. Collectively, these findings reinforce the importance of incorporating thermal tolerance and local adaptation criteria into *Trichogramma* strain selection for inundative release programs. The use of autochthonous, heat-adapted populations is expected to improve establishment, parasitism efficiency, and overall field performance under high-temperature conditions.

Future research should prioritize field validation of *Trichogramma* performance under naturally fluctuating temperatures to confirm laboratory findings and refine biological control strategies, considering that all results of the present study were obtained under constant-temperature conditions. Additionally, exploring physiological acclimation and the selection of locally adapted populations could provide practical approaches to enhancing thermal resilience, thereby improving the efficacy of *Trichogramma* releases under current and predicted climate conditions.

## Data Availability

The original contributions presented in the study are included in the article, further inquiries can be directed to the corresponding authors.
